# Internet addiction and psychological distress in highly schizotypal students

**DOI:** 10.1192/j.eurpsy.2023.617

**Published:** 2023-07-19

**Authors:** Y. Boukadida, F. fekih_romdhane, R. Away, R. Damak, F. ghrissi, M. Cheour

**Affiliations:** Department of Psychiatry Ibn Omrane, razi hospital, tunis, Tunisia

## Abstract

**Introduction:**

There is some limited evidence of an association between technology addictions and the emergence of pre-psychotic symptoms, high psychoticism , psychotic like experiences, and high schizotypy among young non-clinical adults. These addictions and their subsequent distress are likely to contribute to transition to psychosis in individuals at risk .

**Objectives:**

we aimed to explore the association between Internet addiction and distress in the high schizotypy group , expecting that the two would be associated .

**Methods:**

From a pool of 700 students, the final sample consisted of 74 low schizotypal and 70 high schizotypal students. Data were collected using a self-administered questionnaire which contained five research scales: The Schizotypal Personality Questionnaire (SPQ) , the Depression, Anxiety and Stress Scales (DASS-21) , the Internet Addiction Test (IAT).

**Results:**

Bivariate analyses revealed significant positive correlations between Internet addiction and depression (r=.344, p<.0001), anxiety (r=.320, p<.0001) and stress (r=.336, p<.0001) in the high schizotypy group. In this same group, positive symptoms of schizotypy correlated positively with internet addiction (r=.294 ;p=.014).

After controlling for demographics, psychosocial factors and schizotypy symptoms, Internet addiction predicted psychological distress in the high schizotypy group (R2= 0.380, F (13, 55) = 2.597, p<0.001) and explained an additional 19.7% of variation in DASS total scores in the final model (R2 Δ= 0.197)

**Conclusions:**

This study’s results portrayed a statistically significant relationship between addiction to Internet and psychological distress in this at risk group.Although preliminary, our findings shed light on relatively new avenues for prevention and early intervention in psychosis. Given the widespread use of Internet among individuals with schizotypy, clinicians and researchers should find ways to utilize it as a potential resource to help these vulnerable individuals in their care pathways, by turning it into a protective rather than stressor factor.Finally, our findings highlight the need for further studies to better understand Internet use patterns and effects on young individuals in order to help mitigate its risks and increase its benefits.

**Disclosure of Interest:**

None Declared

